# 

**Published:** 1919-05-03

**Authors:** 


### The Book Market.

**List of New Books Received from the following publishers:—**

Chapman & Hall: "The Cost of Food. A Study in Dietaries." By Ellen H. Richards and John F. Porter. 5s. net.

G. Bell & Sons: "Inorganic Chemistry." By James Walker, LL.D., F.R.S.

T. Fisher Unwin: "The Thunderbolt." By Geo. Colmore. 7s. net.

Hy. Kimpton: "The Physiological Feeding of Children." By Eric Pritchard. 3s. 6d. net.

**Mr. Fredk. C. E. Stewart, of Dublin, left £2,000 to the Royal Victoria Hospital, Belfast; £500 each to the Forster Green Consumption Hospital, the Samaritan Hospital, the Children's Hospital, the Royal Ulster Eye and Throat Hospital, and the Hospital for Nervous Diseases, all of Belfast.**

### PASSING EVENTS AND TOPICS.

Sir Kingsley Wood, M.P., states that in London there are eight doctors and nine partnerships, each with over 5,000 insured persons on their panels.

Sir Arthur Newsholme states that, during the last 11 weeks of 1918, in 96 great towns in the country 44,537 deaths were recorded as due to influenza.

£658 was collected on the occasion of the annual service in St. Chad's Church, Shrewsbury, in aid of the Royal Salop Infirmary, as compared with £500 last year.

Within the last twelve months 100 additional hospitals in the United States have established clinics for the treatment of venereal disease.

Devizes and Pewsey Joint Isolation Hospital Committee, after providing for the needs of the hospital, made a profit last year of £64 on potatoes grown. The previous year it raised corn, which involved a loss of £3.

A scheme is afoot at Bedlington Station to build a hospital as a war memorial.

It is stated that in New York only one person in ten needing hospital treatment gets it. For contagious diseases alone London had before the war six times as many hospital beds as New York.

Eton boys, in the past four years, have subscribed £430, and the masters £1,444, to the Public Schools Hospital Fund. For the same object, the Old Etonian Association has collected £1,218.

The London County Council states that Orders have been made under Part IV. (Lying-in Homes) of the London County Council (General Powers) Act, 1915, (1) cancelling the registration of Mrs. Annie Hart, 4 Belgrave Terrace, Brixton, and (2) refusing to register the name of Mrs. Matilda Hart, 19 Caxton Road, Shepherd's Bush.

### WAXOL.

We have tested this preparation and find it satisfactory. We tried it on a somewhat neglected table and the surface showed capital results afterwards with a good polish restored. It is easy to use, and a little goes a long way. Being antiseptic it is very suitable for institution use, both for polishing floors and furniture. The makers are Cuxson, Gerrard & Co., Crown Buildings, Birmingham. The price is 1s. 8d. lb., with a reduction on large quantities.

